# Thyroid hormones and thyroid hormone sensitivity indices correlate with volumetric bone mineral density and vertebral fracture risk among the osteoporotic population in northern China

**DOI:** 10.3389/fendo.2026.1785651

**Published:** 2026-06-04

**Authors:** Zhou Meicen, Deng Wei, Wang Yanai, Huo Lili, Gao Tan, Chen Jia

**Affiliations:** Department of Endocrinology, Beijing Jishuitan Hospital, Capital Medical University, Beijing, China

**Keywords:** bone density, fracture risk, osteoporosis, thyroid hormone, thyroid hormone sensitivity index

## Abstract

**Objective:**

Thyroid hormones play critical roles in regulating bone turnover and maintaining bone mass. However, the relationship among thyroid hormones, their sensitivity indices, bone mineral density (BMD) and fracture risk remained unclear in the Chinese osteoporotic population. This study aimed to investigate the associations between thyroid hormones, their sensitivity indices, and both volumetric BMD (measured by quantitative computed tomography, QCT-BMD), areal BMD (measured by dual-energy X-ray absorptiometry, DXA BMD), as well as the risk of vertebral fractures, among primary osteoporotic individuals aged over 50 years old of euthyroid adults in northern China (north of the Yellow River).

**Methods:**

A total of 1, 386 men and postmenopausal women (aged ≥50 years) with euthyroidism and primary osteoporosis were recruited from Beijing Jishuitan Hospital during January 2021 to December 2024. Participants were divided into two groups: those without vertebral fractures (n = 856) and those with vertebral fractures (n = 530). Demographic characteristics, serum bone turnover markers (ALP, tP1NP, β-CTX, OC, PTH), thyroid hormones (TT4, TT3, TSH, FT3, FT4), thyroid hormone sensitivity indices (FT4/FT3, TSHI, TT4RI, TFQI), lumbar QCT-BMD, lumbar DXA-BMD, and total hip DXA-BMD were compared between groups. Univariate and multivariate logistic regression analyses, restricted cubic spline (RCS) regression, and subgroup analyses were performed to evaluate the independent effects of thyroid hormones and their sensitivity indices on BMD measures and vertebral fracture risk, adjusting for potential confounders including age, sex, BMI, smoking, alcohol consumption, diabetes, hypertension, and hyperlipidemia.

**Results:**

The vertebral fracture group exhibited significantly higher TT3 levels compared to the non-fracture group (TT3: 1.98 [1.60, 2.89] vs. 1.76 [1.50, 1.91] nmol/L, *P* < 0.001), while TT4 levels were significantly lower than the non-fracture group (TT4: 89.50 [39.65, 101.00] vs. 103.00 [85.60, 115.00] nmol/L, *P* < 0.001). No significant difference was observed for TSHI, TT4RI, or TFQI between groups. After multivariable adjustment, elevated TT3 remained independently associated with increased lumbar fracture risk (OR (95% CI): 1.02 [1.01–1.04], *P* = 0.018), the positive relationship remained significant in RCS analysis (*P* < 0.001). TSH showed a significant positive association with lumbar QCT-BMD (β (95% CI): 0.89 [0.07–1.71], *P* = 0.034) and in RCS analysis(*P* = 0.013). In contrast, FT4/FT3 and FT3 were positively associated with lumbar QCT-BMD in the adjusted logistic regression (β (95% CI): 12.08 [6.18~17.98], *P* < 0.001; β (95%CI):3.69 (0.31 ~ 7.07), *P* = 0.033), but exhibited a nonlinear and negative association with lumbar QCT-BMD in RCS analysis (*P* = 0.003, *P* = 0.008). Subgroup analyses stratified by gender, age, BMI, diabetes, hypertension, hyperlipidemia, cardiovascular disease, smoking, and alcohol drinking revealed significant interaction effects between FT4/FT3 and lumbar QCT-BMD in gender-specific (*P*-interaction = 0.046) and age-specific analysis (*P*-interaction = 0.021). Notably, in individuals under 60 years old, higher FT4/FT3 was associated with increased lumbar QCT-BMD(β (95% CI): 10.62 [2.84–18.40], *P* = 0.009).

**Conclusion:**

Our findings demonstrated that thyroid hormones—specifically FT3, TT3, TSH—and the peripheral thyroid hormone sensitivity index FT4/FT3 were more strongly associated with vertebral fracture risk and bone mineral density. Notably, these associations were more pronounced with vBMD measured by QCT. Collectively, these results highlighted the potential role of FT3, TT3, TSH, and the peripheral thyroid sensitivity index FT4/FT3 as indicators of bone mass and fracture risk prediction in this population.

## 1 Introduction

Osteoporosis is a systemic skeletal disorder characterized by reduced bone mass and deteriorative bone microarchitecture, leading to a higher risk of fractures (1). The most severe clinical consequence of this condition is osteoporotic fracture (2). Epidemiological studies demonstrated that each 10% reduction in bone mineral density (BMD) was associated with a 2 to 3 fold increase in fracture risk. With the accelerating global aging of the population, osteoporosis has emerged as a major public health challenge, imposing substantial burden on healthcare systems (3–5).

Osteoporosis is closely associated with age, sex, hormones and other factors. Accumulating evidence indicated a significant relationship between thyroid hormones and bone metabolism, and thyroid dysfunction contributed to alterations in bone structure. In hyperthyroidism, accelerated bone turnover leads to loss of bone mass and an increased risk of fragility fractures, accompanied by elevated bone turnover markers both in bone resorption and formation (6). Subclinical hyperthyroidism increased fracture risk by 36% compared to euthyroidism (7). However, the precise role of thyroid hormones in osteoporosis remained controversial. In TSH receptor (TSHR) knockout mouse models, elevated TSH levels were paradoxically associated with decreased bone mineral density (BMD) (8). In contrast, a meta-analysis in postmenopausal women demonstrated a significant positive correlation between serum TSH and BMD, suggesting that higher TSH levels within the normal reference range (e.g., >2.5 mIU/L) might be protective against osteoporosis (5).

In recent years, Laclaustra et al. proposed a formula to calculate the thyroid hormone sensitivity index, known as the Thyroid Feedback Quantification Index (TFQI) (9). Other indices reflecting thyroid hormone sensitivity included the Total Thyroxine Resistance Index (TT4RI), Thyrotropin Sensitivity Index (TSHI), Principal Component-Based Thyroid Feedback Quantification Index (PTFQI), FT3/FT4 ratio, and FT4/FT3 ratio. These markers provided insights into the functional status of the hypothalamic-pituitary-thyroid (HPT) axis (10–12). Accumulating evidence indicated that impaired thyroid hormone sensitivity was closely associated with various metabolic disorders. Most studies investigating the relationship between thyroid hormone sensitivity and bone mineral density (BMD) or osteoporosis utilized data from the National Health and Nutrition Examination Survey (NHANES). These analyses suggested that reduced thyroid hormone sensitivity was significantly associated with higher lumbar BMD, implying that dysregulation of central and peripheral thyroid hormone feedback mechanisms might contribute to alterations in bone metabolism (13). C. Liu et al. reported that impaired thyroid hormone sensitivity was significantly linked to both osteoporosis and fracture risk (14). Similarly, Zihan Chen et al. demonstrated that decreased thyroid hormone sensitivity was associated with deteriorative bone microstructure among euthyroidism (15).

Dual-energy X-ray absorptiometry (DXA) is the most widely used method for measuring bone mineral density (BMD) and diagnosing osteoporosis currently (16). It provides areal BMD, which reflects a two-dimensional projection of both trabecular and cortical bone density. However, DXA measurements can be influenced by various scanning artifacts, potentially leading to inaccurate results. Factors such as the presence of osteophytes, aortic calcification, and tall stature may cause overestimation of BMD due to increased projected bone area or surrounding calcified structures (17). In addition to DXA, quantitative computed tomography (QCT) is another commonly used modality for assessing bone density. QCT measures volumetric BMD (vBMD), specifically within trabecular bone, and expresses results in milligrams of hydroxyapatite per cubic centimeter (mg/cm³). The key advantage of QCT is that it is less affected by bone size, cortical bone overlap, or surrounding soft tissues—such as adipose tissue, making it a more accurate tool for evaluating the trabecular bone density (18). To date, most studies have focused on the relationship between thyroid hormone sensitivity and DXA-derived areal BMD, with limited evidence available on its association with QCT-measured volumetric BMD.

This study investigates the associations between thyroid hormones, their sensitivity indices, and both volumetric (QCT-BMD), areal (DXA BMD) bone density measures, as well as vertebral fracture risk among primary osteoporotic individuals of euthyroidism in northern China (north of the Yellow River).

## 2 Subjects and methods

### 2.1 Subjects

A total of 1, 386 participants were enrolled from the department of endocrinology in Beijing Jishuitan Hospital between January 2021 and December 2024. All subjects were aged 50 years or older, resided in northern China (north of the Yellow River), and had normal thyroid function. The participants included both postmenopausal women and men diagnosed with primary osteoporosis according to the criteria of the World Health Organization (WHO) [1994]: osteoporosis based on dual-energy X-ray Absorptiometry (DXA) was defined as a T-score ≤ –2.5 standard deviations (SD), osteopenia as a T-score between –1.0 and –2.5 SD, and normal bone mass as a T-score ≥ –1.0 SD. Participants had not initiated any anti-osteoporosis pharmacotherapy prior to enrollment, including anti-resorptive or anabolic agents, and were not regularly taking calcium supplements or calcitriol. Exclusion criteria included severe cardiovascular(acute and chronic heart failure (NYHA class III, IV) or with unstable vital signs), hepatic(decompensated liver cirrhosis), renal(acute and chronic renal failure (eGFR ≤30ml/min)), pulmonary(respiratory failure I、II), or neurological diseases, autoimmune disorders, hypopituitarism, and current use of thyroid hormones, antithyroid drugs, glucocorticoids, immunosuppressants, or sex hormone replacement therapy. Smokers (Cigarettes) refer to individuals who have smoked continuously or cumulatively for six month or more in their lifetime. Drinkers(Alcohol)refer to individuals who consume more than 5g of alcohol daily and more than 3 times a week.

### 2.2 Definition of vertebral fracture

In the present study, the enrolled patients with vertebral fractures were those diagnosed with osteoporotic compression fractures of the thoracic or lumbar spine occurring within the preceding six months. All participants underwent lateral X-ray and CT imaging of the thoracic and lumbar spine. Image interpretation was performed by radiologists in a blinded manner. The severity of compression fractures was assessed and classified according to the Genant grading system based on the X-ray findings, and patients with Genant grades 1, 2, and 3 were included in the study cohort.

### 2.3 Bone mineral density measurement

#### 2.3.1 Dual-energy X-ray absorptiometry

Lumbar spine (L1–L4) and right hip bone mineral density (BMD) were measured by DXA using GE Lunar Prodigy DXA scanners (GE Lunar Prodigy and DPX Bravo DXA scanners, GE Healthcare, WI, USA). Vertebrae with significant thoracolumbarbursting fractures or structural deformities were manually excluded from analysis. The right hip BMD assessments included three regions: the femoral neck, greater trochanter, and Ward’s triangle.

#### 2.3.2 Quantitative computed tomography

QCT scans were performed using a Toshiba Aquilion 64-slice spiral CT scanner (Toshiba, Tokyo, Japan), with a Mindways 5-sample solid-state calibration phantom (Mindways, Austin, TX, USA) for standardized calibration. Raw scan data were analyzed using Mindways QCT Pro software (Mindways Software, Inc., Austin, TX, USA), which applies phantom-based calibration to derive volumetric BMD values. Measurements were obtained at vertebral levels L1–L4 and adjacent vertebrae. The regions of interest (ROIs) were placed in the central portion of each vertebral body (L2–L4), carefully avoiding cortical bones and the basivertebral vascular plexus. BMD classification followed established guidelines from the International Society for Clinical Densitometry (ISCD) and the American College of Radiology lumbar spine QCT cutoffs (14, 15): osteoporosis (<80 mg/cm³), osteopenia (80–120 mg/cm³), and normal bone mass (>120 mg/cm³) (19).

### 2.4 Thyroid hormone measurement

Fasting venous blood samples were collected, and serum was separated for laboratory analysis. The following parameters were measured: thyroid hormones—total thyroxine (TT4), total triiodothyronine (TT3), thyroid-stimulating hormone (TSH), free thyroxine (FT4), and free triiodothyronine (FT3) and 25-hydroxyvitamin D3 [25-(OH)VD3]. All assays were conducted using the Roche cobas 8000 e 801 fully automated chemiluminescent immunoassay analyzer (Roche Diagnostics, Switzerland) based on the electrochemiluminescence method. Reference ranges were as follows: TT3 (0.77–3.1 nmol/L), TT4 (64.3–189.6 nmol/L), TSH (0.270–4.200 mIU/L), FT4 (12.0–22.0 pmol/L), and FT3 (3.1–6.8 pmol/L).

### 2.5 Calculation method of thyroid hormone sensitivity index

The thyroid hormone sensitivity index was calculated based on formulas proposed by Laclaustra et al. Central sensitivity parameters included: the Thyroid Feedback Quantile-based Index (TFQI) = cdfFT4 – (1 – cdfTSH) (9); the Thyrotropin-Thyroxine Resistance Index (TT4RI) = FT4 × TSH (6); and the Thyroid Stimulating Hormone Index (TSHI) = ln TSH + 0.1345 × FT4 (11). Peripheral sensitivity was assessed using the free thyroxine to free triiodothyronine ratio (FT4/FT3) = FT4 ÷ FT3 (8). Higher values of TFQI, TSHI, TT4RI, and FT4/FT3 indicate reduced tissue sensitivity to thyroid hormones.

### 2.6 Biochemical index measurement

Serum calcium (Ca), phosphorus (P), total cholesterol (TC), alkaline phosphatase (ALP), triglycerides (TG), and low-density lipoprotein cholesterol (LDL-C) were measured using a Hitachi automated biochemical analyzer. Serum total procollagen type I N-terminal propeptide (tP1NP), β-isomerized C-terminal telopeptide of type I collagen (β-CTX), osteocalcin (OC), 25-hydroxyvitamin D3 [25-(OH)VD3], and parathyroid hormone (PTH) were quantified using the Roche electrochemiluminescence immunoassay system (Roche Diagnostics, Switzerland) via the electrochemiluminescence immunoassay method.

### 2.7 Statistical methods

This study was designed as an observational cross-sectional study, with multiple linear regression and binary logistic regression employed as the primary analytical methods. Sample size estimation was conducted in accordance with the principles of *a priori* power analysis for multiple regression models, taking into account the study design, the number of variables to be included, and established practices in prior literature of similar scope. Following the inclusion of key exposure variables and multiple covariates in the final adjusted model, and using conventional parameters for calculation, the minimum required sample size was determined to be approximately 118 participants. The actual sample size recruited in this study meets the statistical power requirements for the intended analyses.

Group comparisons were conducted between the non-fracture and vertebral fracture groups. Continuous variables with normal distribution were expressed as mean ± standard deviation ( $\overset{-}{x}$ ± s) and compared using independent samples t-tests. Non-normally distributed continuous variables were presented as median (interquartile range) [M (P25, P75)] and analyzed using the Mann–Whitney U test. Categorical variables were summarized as frequency (percentage) [n (%)] and compared using the chi-square test; For continuous outcome variables associated with BMD, including QCT-BMD, vertebral DEXA-BMD, and total hip DEXA-BMD, linear regression models were employed to perform association analyses. For the binary outcome of vertebral fracture, a binary logistic regression model was used for analysis. Restricted cubic spline (RCS) regression was employed to explore potential non-linear relationships. Subgroup analysis was conducted to evaluate relationships by stratifying sex, age, BMI, diabetes, hypertension, hyperlipidemia, smoking, and alcohol consumption. All statistical analyses were performed using R software (version 4.3.2). A two-sided *P*-value < 0.05 was considered statistically significant.

## 3 Discussion

This cross-sectional study included postmenopausal women and men aged over 50 years old among primary osteoporotic individuals of euthyroidism in northern China. We investigated the associations between thyroid hormones, thyroid sensitivity indices, and both volumetric bone mineral density (QCT-BMD) and areal bone mineral density (DXA BMD), as well as the risk of vertebral fractures. Our findings indicated that, in this population, TT3, FT3, and the peripheral thyroid hormone sensitivity index FT4/FT3 were more strongly associated with volumetric BMD (QCT-BMD) and vertebral fracture risk. FT4/FT3 and FT3 were positively associated with lumbar QCT-BMD in the adjusted logistic regression, but exhibited a nonlinear and negative association with lumbar QCT-BMD in RCS analysis. The results derived from the linear regression model and the RCS analysis were not inherently contradictory. Specifically, the multivariable linear regression analysis revealed the overall average trend of the association between exposure and outcome, whereas the RCS analysis further demonstrates that this trend may exhibit nonlinear variations within specific local intervals. These two analytical approaches were not mutually exclusive; rather, they complemented each other by depicting the exposure-outcome relationship from distinct methodological perspectives, thereby providing a more comprehensive understanding of the underlying association. Elevated TT3 levels were positively associated with increased risk of vertebral fractures. In contrast, TSH showed a significant positive association with lumbar QCT-BMD.

Previous studies have indicated that areal bone mineral density (aBMD) measured by DXA was more susceptible to be influenced by demography compared to volumetric bone mineral density (vBMD) assessed by QCT (17, 20, 21). In the univariate analysis of this study, the FT4/FT3 ratio were significantly negatively associated with total hip aBMD, whereas FT3 showed a significant positive association. However, after adjustment in multivariable regression models and comprehensive evaluation using restricted cubic spline (RCS) analysis, no statistically significant association was observed between these thyroid parameters and DXA-derived aBMD. This suggested that aBMD may be influenced by multiple confounding factors, while vBMD obtained by QCT appeared to be more stable and less prone to external interferences (20, 21). In this study, both aBMD and vBMD were employed to evaluate bone mass. Notably, in RCS analysis, FT3 and the FT4/FT3 ratio were significantly inversely associated with lumbar QCT-BMD, and TSH was positively associated with it. In contrast, no more significant association was detected with DXA-BMD, indicating that thyroid hormones and their sensitivity indices were more strongly correlated with vBMD than with aBMD.

Thyroid hormones include triiodothyronine (T3), thyroxine (T4), free T3 (FT3), free T4 (FT4), and thyroid-stimulating hormone (TSH). T3 and T4 are secreted into the bloodstream, where the majority bind to thyroxine-binding globulin, while only a small fraction remains in the biologically active free form (FT3 and FT4), which mediates physiological effects in tissues (22). T4 is peripherally converted to the more potent T3, which enters the cell nucleus and activates thyroid hormone receptors α or β (TRα and TRβ). TRα is the predominant isoform expressed in bone tissue (23). Thyroid hormones play a critical role in regulating bone turnover. Hyperthyroidism induced a state of high bone turnover, accelerating bone loss and increasing the risk of osteoporosis and hip fractures (24). Long-term administration of thyroid hormone at suppressive doses might reduce bone density and elevate the fracture risk, particularly in postmenopausal women, whereas these effects were less evident in premenopausal individuals (6). Conversely, hypothyroidism reduced bone turnover by suppressing both osteoclastic resorption and osteoblastic activity (6). In populations with normal or lower bone mass, TT4 levels were negatively associated with BMD, and elevated FT4 levels were linked to an increased risk of prior fractures (7). Evidences also suggested that higher FT4 levels within the euthyroid range and lower TSH concentrations were associated with greater hip fracture risk (25). This study found that TSH was significantly positively correlated with vertebral QCT-BMD, which was consistent with the findings of a previous systematic review focusing on postmenopausal women with osteoporosis (5). However, a cross-sectional study conducted among euthyroid populations failed to identify a significant association between TSH and BMD (7), while an umbrella review indicated that reduced TSH levels served as an independent risk factor for decreased bone mineral density (26). Therefore, the relationship between TSH and BMD remained controversial, and such inconsistent conclusions may be attributed to differences in study populations and research designs. In the present study based on the osteoporosis cohort, TT3 levels were closely linked to an increased risk of vertebral fracture, and FT3 showed a significant negative correlation with vertebral QCT-BMD, while a significant positive correlation between FT3 and BMD has been reported in euthyroid patients with type 2 diabetes mellitus (T2DM) (27). Collectively, the correlations of TSH, TT3 and FT3 with BMD and fracture risk in our study population suggested that the regulatory effects of thyroid hormones on bone turnover might not be mediated by the single thyroid hormone. Instead, TSH, FT3 and TT3 were all involved in the regulation of bone metabolism and turnover.

In recent years, increased attention has been attracted toward the association between thyroid hormone sensitivity indices and bone mineral density. Shuai Chen et al., using data from the NHANES database, reported that reduced thyroid hormone sensitivity was associated with higher lumbar BMD (28). Qilin Wang, et al. demonstrated that elevated FT3/FT4 ratio and TPOAb levels were linked to increased vertebral bone mass and a higher risk of vertebral fractures (25). Xuelun Wu et al. identified a U-shaped association between thyroid hormone sensitivity indices and the risk of osteoporosis in individuals with type 2 diabetes (29). However, studies focusing on Chinese populations remained limited. The present study enrolled individuals from northern China and conducted both vBMD and aBMD to assess bone density comprehensively. Our findings did not reveal a significant association between central thyroid hormone sensitivity indices and either BMD or vertebral fracture risk. In contrast, the peripheral thyroid hormone sensitivity index FT4/FT3 was significantly inversely associated with lumbar QCT-BMD. Liu C, et al. also found significant positive correlations between FT3/FT4 and BMD in a cohort of euthyroid adults in the United States (14). In addition, we further performed subgroup analyses in this study. The subgroup analyses revealed significant interactive effects of gender and age on the associations between FT4/FT3 and vertebral QCT-BMD. After age stratification, the results indicated that among individuals aged under 60 years, FT4 and FT3 exerted a more pronounced impact on vertebral QCT-BMD.

Metabolic disorder, including hyperglycemia, dyslipidemia, and hypertension, were known to affect the bones. Patients with diabetes exhibited reduced BMD, and in this study, after adjusting for potential confounders, diabetes was independently associated with an increased risk of vertebral fractures (30). Though the relationship between lipid profiles and bone metabolism remains controversial, in our analysis total cholesterol was significantly negatively correlated with lumbar DXA-BMD and total hip DXA-BMD, indicating that higher total cholesterol levels were associated with lower aBMD. Similarly, LDL-C was negatively associated with total hip DXA-BMD, suggesting a detrimental effect of elevated LDL-C on hip bone density. Nevertheless, no significant associations were observed between the previous history of hyperlipidemia and either BMD measures or vertebral fracture risk, implying that circulating lipid levels may have a more direct metabolic impact than previous hyperlipidemia history.

In summary, among euthyroid individuals over 50 years old with primary osteoporosis in northern China, our findings demonstrated that thyroid hormones—specifically FT3, TT3, TSH—and the peripheral thyroid hormone sensitivity index FT4/FT3 were more strongly associated with vertebral fracture risk and bone mineral density. Notably, these associations were more pronounced with vBMD measured by QCT. FT3 and FT4/FT3 were significantly positively associated with lumbar QCT-BMD in the multivariate regression analysis but inversely correlated with lumbar QCT-BMD in RCS analysis. Elevated TT3 levels were independently linked to an increased risk of vertebral fractures. In contrast, TSH showed a significant positive association with lumbar QCT-BMD. Collectively, these results highlighted the potential role of FT3, TT3, TSH, and the peripheral thyroid sensitivity index FT4/FT3 as indicators of bone mass and fracture risk prediction in this population.

### 3.1 Limitations of the study

This study was designed as a single-center retrospective study, with the study population restricted to patients with osteoporosis who were treated in the Department of Endocrinology, Beijing Jishuitan Hospital. Furthermore, the study population did not include fracture patients with Genant grade 4, which may artificially increase the DXA-BMD values in the fracture group and weaken the observed association between DXA bone mineral density and fracture risk. Consequently, a certain degree of selection bias may exist, which could potentially affect the external validity of the study results. The results of this study might provide appropriate references for broader population-based studies in our following research.

### 3.2 Baseline characteristics

A total of 1, 386 participants were recruited in this study, including 856 individuals without vertebral fractures and 530 with vertebral fractures. The prevalence of vertebral fractures in the cohort was 38.24% (530/1, 386). Baseline comparisons between the two groups revealed that the vertebral fracture group had significantly higher levels of alkaline phosphatase (ALP), total cholesterol (TC) and total triiodothyronine (TT3) compared to the non-fracture group (vertebral fracture vs. non-fracture: ALP: 80.00 [67.00, 95.50] vs. 75.00 [60.00, 91.00] U/L, *P* = 0.044; TC: 5.08 [4.31, 5.70] vs. 4.76 [3.98, 5.51] mmol/L, *P* = 0.007; TT3: 1.98 [1.60, 2.89] vs. 1.76 [1.50, 1.91] nmol/L, *P* < 0.001, **Table 1**). In contrast, serum 25-(OH)VD3, lumbar QCT-BMD and TT4 were significantly lower in the vertebral fracture group (vertebral fracture vs. non-fracture: 25-(OH)VD3: 21.20 [15.45, 28.00] vs. 23.43 [17.55, 30.01] μg/L, *P* = 0.025; QCT-BMD: 58.30 [41.80, 75.30] vs. 72.95 [55.25, 88.27] mg/cm³, *P* < 0.001; TT4: 89.50 [39.65, 101.00] vs. 103.00 [85.60, 115.00] nmol/L, *P* < 0.001, **Table 1**).

**Table 1:** Baseline characteristics and comparative analysis between individuals with and without vertebral fractures.

Variables	Overall (n = 1386)	Without vertebral fracture (n = 856)	With vertebral fracture (n = 530)	t/Z/χ²	*P*
Age(years)	66.27 ± 9.64	65.87 ± 10.49	66.76 ± 8.45	-0.96	0.336
BMI kg/m^2^	23.45 ± 3.38	23.29 ± 3.42	23.65 ± 3.32	-1.11	0.269
TT3 nmol/L	1.84 (1.50, 2.00)	1.76 (1.50, 1.91)	1.98 (1.60, 2.89)	-2.88	<0.001
TT4 nmol/L	95.46 (34.60, 113.60)	103.00 (85.60, 115.00)	89.50 (39.65, 101.00)	-5.12	<0.001
TSH mIU/L	2.18 (1.45, 3.32)	2.21 (1.46, 3.28)	2.16 (1.44, 3.51)	-0.43	0.664
FT4 pmol/L	16.47 ± 2.63	16.39 ± 2.69	16.56 ± 2.56	-0.68	0.497
FT3 pmol/L	4.56 ± 0.99	4.57 ± 1.23	4.56 ± 0.58	0.10	0.919
TC mmol/L	4.89 (4.11, 5.56)	4.76 (3.98, 5.51)	5.08 (4.31, 5.70)	-2.71	0.007
TG mmol/L	1.22 (0.88, 1.80)	1.17 (0.88, 1.65)	1.32 (0.92, 1.84)	-1.55	0.120
LDL-C mmol/L	2.68 (2.17, 3.29)	2.62 (2.12, 3.29)	2.70 (2.20, 3.28)	-0.42	0.672
Ca mmol/L	2.32 ± 0.13	2.32 ± 0.14	2.33 ± 0.10	-1.10	0.273
IP mmol/L	1.07 ± 0.16	1.06 ± 0.17	1.08 ± 0.14	-1.48	0.139
ALP IU/L	77.00 (63.00, 95.00)	75.00 (60.00, 91.00)	80.00 (67.00, 95.50)	-2.02	0.044
tP1NP ng/ml	46.70 (32.00, 64.00)	47.23 (33.42, 63.88)	46.60 (31.30, 64.30)	-0.06	0.949
β-CTX ng/ml	0.38 (0.21, 0.54)	0.39 (0.23, 0.57)	0.37 (0.17, 0.53)	-1.25	0.211
OC ng/ml	15.60 (11.20, 21.40)	15.40 (10.90, 21.55)	15.70 (11.80, 21.40)	-0.48	0.628
PTH pg/ml	35.10 (26.50, 46.90)	36.40 (26.72, 47.27)	33.80 (25.95, 45.25)	-1.25	0.210
25(OH)VD3 ng/ml	22.06 (16.47, 28.80)	23.43 (17.55, 30.01)	21.20 (15.45, 28.00)	-2.24	0.025
Lumbar QCT BMD mg/cc	65.00 (47.20, 83.20)	72.95 (55.25, 88.27)	58.30 (41.80, 75.30)	-5.72	<0.001
Lumbar DEXA-BMD mg/cm2	0.79 (0.69, 0.90)	0.81 (0.73, 0.90)	0.79 (0.69, 0.89)	-0.30	0.764
Hip DEXA-BMD mg/cm2	0.73 (0.65, 0.80)	0.74 (0.65, 0.80)	0.72 (0.65, 0.80)	-0.85	0.398
TSHI	3.02 (2.59, 3.42)	3.00 (2.57, 3.41)	3.02 (2.60, 3.46)	-0.58	0.561
TT4RI	35.53 (23.60, 54.77)	35.87 (23.00, 53.72)	35.05 (24.08, 56.98)	-0.49	0.624
TFQI	-0.01 (-0.33, 0.34)	0.01 (-0.31, 0.34)	-0.02 (-0.36, 0.35)	-0.25	0.806
FT4/FT3	3.71 ± 0.77	3.72 ± 0.82	3.69 ± 0.70	0.51	0.611
Gender, n(%)				3.12	0.077
Female	701 (50.58)	472(55.14)	229(43.21)		
Male	685 (49.42)	384 (44.86)	301 (56.79)		
Cigarettes, n(%)				3.12	0.077
No	97 (7.00)	76 (8.88)	24 (4.53)		
Yes	1289 (93.00)	780 (91.12)	506 (95.47)		
Alcohol, n(%)				2.49	0.115
No	50 (3.61)	42 (4.91)	11 (2.08)		
Yes	1336 (96.39)	814 (95.09)	519 (97.92)		
Diabetes, n(%)				1.01	0.315
No	418 (30.16)	274 (32.01)	147 (27.74)		
Yes	968 (69.84)	582 (67.99)	383(72.26)		
Hypertension, n(%)				1.15	0.284
No	450 (32.47)	296 (34.58)	158 (29.81)		
Yes	936 (67.53)	560 (65.42)	372 (70.19)		
Hyperlipidemia, n(%)				0.50	0.481
No	415 (29.94)	268 (31.31)	150 (28.30)		
Yes	971 (70.06)	588 (68.69)	380 (71.70)		
Cardiovascular disease, n(%)				0.06	0.811
No	151 (10.89)	90 (10.51)	60 (11.32)		
Yes	1235 (89.11)	766 (89.49)	470 (88.68)		

No significant differences were observed between the two groups in serum calcium, phosphorus, tP1NP, β-CTX, OC or PTH levels (**Table 1**). Similarly, no significant differences were found for thyroid hormone sensitivity indices, including TFQI, TSHI, TT4RI, and FT4/FT3 (all P > 0.05; **Table 1**). With regard to comorbidities and lifestyle factors, the prevalence of diabetes, hypertension, cardiovascular disease, hyperlipidemia, smoking, and alcohol consumption did not differ significantly between groups (**Table 1**).

### 3.3 Univariate regression analysis of bone turnover markers, thyroid hormones, and their sensitivity indices with lumbar QCT-BMD, DXA-BMD, and vertebral fracture risk

Among bone turnover markers, the bone formation indicators –ALP, tP1NP showed significant negative associations with both lumbar QCT-BMD and total hip DXA-BMD, respectively. Specifically, ALP, tP1NP was negatively associated with lumbar QCT-BMD (ALP: β (95% CI): -0.11 [-0.19, -0.04], *P* = 0.003; tP1NP: -0.08(-0.16, -0.01), *P* = 0.023) and total hip DXA-BMD (ALP: β (95% CI): -0.01 [-0.99, -0.01], *P* < 0.001; tP1NP: -0.01[-0.99, -0.01], *P* < 0.001). OC also exhibited a significant negative association with total hip DXA-BMD (β (95% CI): -0.01 [-0.99, -0.01], *P* < 0.001). Among resorption markers, β-CTX was significantly positively associated with total hip DXA-BMD (β (95% CI): 0.01 [0.01, 0.01], *P* = 0.004). Serum phosphorus (P) was positively associated with both lumbar DXA-BMD (β (95% CI): 2.02 [0.69, 3.34], *P* = 0.003) and total hip DXA-BMD (β (95% CI): 0.08 [0.01, 0.15], *P* = 0.031). Increased 25-(OH)VD3 was associated with a reduced risk of vertebral fractures (OR (95% CI): 0.37 [0.17, 0.80], *P* = 0.032).

Among thyroid hormones, TT3 was significantly negatively associated with total hip DXA-BMD (β (95% CI): -0.01 [-0.99, -0.01], *P* = 0.035), and elevated TT3 or TT4 levels were linked to an increased risk of vertebral fractures (TT3: OR (95% CI): 1.02 [1.01, 1.02], *P* < 0.001; TT4: OR (95% CI): 1.98 [1.32, 2.29], *P* < 0.001). FT3 showed a significant positive association with total hip DXA-BMD (β (95% CI): 0.01 [0.01, 0.03], *P* = 0.012). The peripheral thyroid hormone sensitivity index FT4/FT3 was significantly negatively associated with total hip DXA-BMD (β (95% CI): -0.02 [-0.03, -0.01], *P* = 0.007). In contrast, central sensitivity indices—including the TFQI, TT4RI, and TSHI —showed no statistically significant associations with lumbar QCT-BMD, DXA-BMD at either site, or vertebral fracture risk (**Table 2**).

**Table 2:** Univariate regression analysis of bone turnover markers, thyroid hormones, and their sensitivity indices in relation to lumbar QCT-BMD, DXA-BMD, and vertebral fracture risk.

	Lumbar QCT-BMD		DXA-BMD		Hip DXA-BMD		Vertebral fracture	
Variables	β (95%CI)	*P*	β (95%CI)	*P*	β (95%CI)	*P*	OR (95%CI)	*P*
Gender								
Female	Ref.		Ref.		Ref.		1.00 (Reference)	
Male	14.51 (6.58 ~ 22.43)	<0.001	-0.01 (-0.69 ~ 0.67)	0.969	0.08 (0.04 ~ 0.11)	<0.001	0.35 (0.17 ~ 0.70)	0.003
Cigarettes								
No	Ref.		Ref.		Ref.		1.00 (Reference)	
Yes	-2.52 (-12.22 ~ 7.17)	0.610	0.05 (-0.77 ~ 0.87)	0.908	-0.09 (-0.13 ~ -0.04)	<0.001	2.03 (0.91 ~ 4.52)	0.083
Alcohol								
No	Ref.		Ref.		Ref.		1.00 (Reference)	
Yes	-11.16 (-24.38 ~ 2.06)	0.099	-0.04 (-1.16 ~ 1.08)	0.945	-0.11 (-0.17 ~ -0.05)	<0.001	2.45 (0.78 ~ 7.72)	0.126
Diabetes								
No	Ref.		Ref.		Ref.		1.00 (Reference)	
Yes	0.61 (-4.79 ~ 6.01)	0.825	0.07 (-0.39 ~ 0.53)	0.766	-0.03 (-0.06 ~ -0.01)	0.007	1.24 (0.82 ~ 1.87)	0.315
Hypertension								
No	Ref.		Ref.		Ref.		1.00 (Reference)	
Yes	0.78 (-4.52 ~ 6.07)	0.774	0.09 (-0.36 ~ 0.54)	0.692	-0.03 (-0.05 ~ -0.01)	0.035	1.25 (0.83 ~ 1.87)	0.284
Hyperlipidemia								
No	Ref.		Ref.		Ref.		1.00 (Reference)	
Yes	0.68 (-4.74 ~ 6.09)	0.807	-0.40 (-0.86 ~ 0.05)	0.084	-0.04 (-0.06 ~ -0.02)	0.001	1.16 (0.77 ~ 1.75)	0.481
Cardiovascular disease								
No	Ref.		Ref.		Ref.		1.00 (Reference)	
Yes	4.77 (-3.18 ~ 12.72)	0.24	0.06 (-0.61 ~ 0.74)	0.855	-0.02 (-0.06 ~ 0.02)	0.256	0.93 (0.51 ~ 1.70)	0.811
Age(years)	-1.21 (-1.44 ~ -0.98)	<0.001	0.01 (-0.01 ~ 0.03)	0.465	-0.01 (-0.99~ -0.01)	<0.001	1.01 (0.99 ~ 1.03)	0.336
BMI kg/m2	-1.64 (-2.36 ~ -0.93)	<0.001	-0.01 (-0.07 ~ 0.05)	0.819	0.01 (0.01 ~ 0.01)	<0.001	1.03 (0.98 ~ 1.09)	0.269
TC mmol/L	2.73 (0.61 ~ 4.85)	0.062	-0.23 (-0.41 ~ -0.05)	0.011	-0.01 (-0.02 ~ -0.01)	0.016	1.26 (1.06 ~ 1.48)	0.007
TG mmol/L	-2.22 (-4.97 ~ 0.53)	0.114	-0.06 (-0.29 ~ 0.18)	0.634	0.01 (-0.00 ~ 0.02)	0.094	1.17 (0.95 ~ 1.45)	0.138
LDL C mmol/L	1.43 (-1.07 ~ 3.93)	0.263	-0.19 (-0.40 ~ 0.02)	0.082	-0.01 (-0.03 ~ -0.01)	0.013	1.03 (0.86 ~ 1.25)	0.727
ALP IU/L	-0.11 (-0.19 ~ -0.04)	0.003	-0.00 (-0.01 ~ 0.00)	0.232	-0.01 (-0.99 ~ -0.01)	<0.001	1.00 (1.00 ~ 1.01)	0.193
Ca mmol/l	7.53 (-12.06 ~ 27.12)	0.452	-1.04 (-2.70 ~ 0.61)	0.217	-0.06 (-0.15 ~ 0.03)	0.201	2.25 (0.50 ~ 10.10)	0.289
IP mmol/L	10.95 (-4.85 ~ 26.75)	0.175	2.02 (0.69 ~ 3.34)	0.003	0.08 (0.01 ~ 0.15)	0.031	2.50 (0.74 ~ 8.41)	0.14
tP1NP ng/ml	-0.08 (-0.16 ~ -0.01)	0.023	-0.00 (-0.01 ~ 0.00)	0.587	-0.01 (-0.99 ~ -0.01)	<0.001	1.00 (0.99 ~ 1.01)	1
β-CTX ng/ml	0.01 (-0.38 ~ 0.40)	0.943	-0.00 (-0.03 ~ 0.03)	0.914	0.01 (0.01 ~ 0.01)	0.004	0.68 (0.34 ~ 1.35)	0.27
OC ng/ml	-0.25 (-0.55 ~ 0.04)	0.091	-0.00 (-0.03 ~ 0.02)	0.708	-0.01 (-0.99 ~ -0.01)	<0.001	1.00 (0.98 ~ 1.02)	0.943
PTH pg/ml	-0.05 (-0.18 ~ 0.07)	0.409	-0.00 (-0.01 ~ 0.01)	0.864	-0.00 (-0.00 ~ 0.00)	0.243	0.99 (0.98 ~ 1.00)	0.144
25-(OH) VD3 ng/ml	0.16 (-0.08 ~ 0.40)	0.188	0.01 (-0.01 ~ 0.03)	0.38	0.00 (-0.00 ~ 0.00)	0.195	0.37 (0.17 ~ 0.80)	0.032
TT4 nmol/l	0.03 (-0.04 ~ 0.09)	0.428	-0.00 (-0.01 ~ 0.01)	0.886	0.00 (-0.00 ~ 0.00)	0.051	1.98 (1.32 ~ 2.29)	<0.001
TT3 nmol/L	-0.01 (-0.07 ~ 0.05)	0.691	-0.00 (-0.01 ~ 0.00)	0.629	-0.01 (-0.99 ~ -0.01)	0.035	1.02 (1.01 ~ 1.02)	<0.001
TSH mIU/L	0.43 (-0.07 ~ 0.93)	0.09	-0.01 (-0.05 ~ 0.04)	0.744	-0.00 (-0.00 ~ 0.00)	0.354	0.99 (0.94 ~ 1.03)	0.602
FT4 pmol L	0.18 (-0.76 ~ 1.13)	0.703	-0.03 (-0.11 ~ 0.05)	0.491	-0.00 (-0.01 ~ 0.00)	0.054	1.03 (0.95 ~ 1.10)	0.496
FT3 pmol/L	0.31 (-2.19 ~ 2.81)	0.806	-0.01 (-0.22 ~ 0.20)	0.935	0.01 (0.01 ~ 0.03)	0.012	0.99 (0.82 ~ 1.20)	0.918
FT4/FT3	0.94 (-2.29 ~ 4.17)	0.57	-0.08 (-0.35 ~ 0.20)	0.576	-0.02 (-0.03 ~ -0.01)	0.007	0.94 (0.73 ~ 1.20)	0.610
TSHI	2.40 (-0.86 ~ 5.67)	0.150	-0.17 (-0.44 ~ 0.11)	0.237	-0.01 (-0.02 ~ 0.01)	0.380	1.10 (0.86 ~ 1.42)	0.452
TT4RI	0.06 (-0.02 ~ 0.13)	0.149	-0.00 (-0.01 ~ 0.00)	0.441	-0.00 (-0.00 ~ 0.00)	0.637	1.00 (1.00 ~ 1.01)	0.698
TFQI	-0.58 (-6.01 ~ 4.84)	0.833	0.05 (-0.40 ~ 0.51)	0.816	-0.01 (-0.03 ~ 0.02)	0.447	0.96 (0.64 ~ 1.45)	0.862

### 3.4 Univariate regression analysis of general demographic characteristics, diabetes, hypertension, hyperlipidemia with lumbar QCT-BMD, DXA-BMD, and vertebral fracture risk

In this population, males exhibited significantly higher lumbar QCT-BMD and total hip DXA-BMD values compared to females (β (95% CI): 14.51 [6.58–22.43], *P* < 0.001; β (95% CI): 0.08 [0.04–0.11], *P* < 0.001) (**Table 2**), and the odds of vertebral fractures were significantly lower in men than in women (OR (95% CI): 0.35 [0.17–0.70], *P* = 0.003) (**Table 2**). Age was negatively associated with both lumbar QCT-BMD and total hip DXA-BMD(β (95% CI): -1.21 [-1.44, -0.98], *P* < 0.001; β (95% CI): -0.01 [-0.99, -0.01], *P* < 0.001) (**Table 2**). BMI showed a significant negative association with lumbar QCT-BMD (β (95% CI): -1.64 [-2.36, -0.93], *P* < 0.001) (**Table 2**), yet demonstrated a significant positive association with total hip DXA-BMD(β (95% CI): 0.01 [0.01–0.01], *P* < 0.001) (**Table 2**). Diabetic patients had lower total hip DXA-BMD compared to non-diabetic participants (β (95% CI): -0.03 [-0.06, -0.01], *P* = 0.007) (**Table 2**). Similarly, individuals with hypertension or hyperlipidemia exhibited reduced total hip DXA-BMD relative to those with normal blood pressure and lipid profiles (β (95% CI): -0.03 [-0.05, -0.01], *P* = 0.035; β (95% CI): -0.04 [-0.06, -0.02], *P* = 0.001) (**Table 2**). Total cholesterol (TC) was inversely associated with both lumbar DXA-BMD and total hip DEXA-BMD, indicating that higher TC levels corresponded to lower bone mineral density (β (95% CI): -0.23 [-0.41, -0.05], *P* = 0.011; β (95% CI): -0.01 [-0.02, -0.01], *P* = 0.016) (**Table 2**). LDL-C was also negatively associated with total hip DXA-BMD (β (95% CI): -0.01 [-0.03, -0.01], *P* = 0.013) (**Table 2**). Additionally, both smoking and alcohol consumption were associated with significantly lower total hip DXA-BMD (β (95% CI): -0.09 [-0.13, -0.04], *P* < 0.001; β (95% CI): -0.11 [-0.17, -0.05], *P* < 0.001) (**Table 2**).

### 3.5 Multivariate regression analysis

In the unadjusted model (Model 1), elevated TT3 was associated with an increased risk of vertebral fractures (OR (95% CI): 1.02 [1.01–1.04], *P* = 0.008), and TSH showed a significant negative association with total hip DXA-BMD (β (95% CI): -0.01 [-0.99, -0.01], *P* = 0.032). In the model adjusted for age, sex, and BMI (Model 2), TT4 was positively associated with lumbar QCT-BMD (β (95% CI): 0.20 [0.04–0.35], *P* = 0.014), and higher TT3 levels remained significantly associated with increased vertebral fracture risks (OR (95% CI): 1.02 [1.01–1.03], *P* = 0.028). TSH was positively correlated with lumbar QCT-BMD (β (95% CI): 1.02 [0.20–1.84], *P* = 0.015) and negatively associated with total hip DXA-BMD (β (95% CI): -0.01 [-0.99, -0.01], *P* = 0.036). FT3 and the FT4/FT3 ratio were both significantly positively associated with lumbar QCT-BMD (FT3: β(95% CI): 3.63 [0.27–7.00], *P* = 0.035; FT4/FT3: β (95% CI): 11.24 [5.50–16.99], *P* < 0.001). Age was inversely associated with both lumbar QCT-BMD and total hip DXA-BMD (Lumbar QCT-BMD: β (95% CI): -1.32 [-1.55, -1.09], *P* < 0.001; total hip DXA-BMD β (95% CI): -0.01 [-0.99, -0.01], *P* < 0.001), while BMI was negatively associated with lumbar QCT-BMD (β (95% CI): -1.54 [-2.19, -0.90], *P* < 0.001) but positively associated with total hip DXA-BMD (β (95% CI): 0.01 [0.01–0.01], *P* < 0.001). Male participants had significantly higher lumbar QCT-BMD and total hip DEXA values compared to females (QCT-BMD: β (95% CI): 13.42 [6.25–20.58], *P* < 0.001; total hip DXA BMD: β (95% CI): 0.06 [0.03–0.10], *P* < 0.001). In the fully adjusted model (Model 3), TT4 remained significantly positively associated with lumbar QCT-BMD (β (95% CI): 0.18 [0.02–0.35], *P* = 0.027). Elevated TT3 continued to be associated with increased risks of vertebral fracture (OR (95% CI): 1.02 [1.01–1.04], *P* = 0.018). TSH demonstrated a significant positive association with lumbar QCT-BMD (β (95% CI): 0.89 [0.07–1.71], *P* = 0.034), while its association with lumbar DXA-BMD was positive but not statistically significant. FT3 was significantly positively associated with lumbar QCT-BMD (β (95% CI): 3.69 [0.31–7.07], *P* = 0.033), and the FT4/FT3 ratio showed a strong positive association (β (95% CI): 12.08 [6.18–17.98], *P* < 0.001). Age was significantly negatively associated with both lumbar QCT-BMD and total hip DXA-BMD (β (95% CI): -1.36 [-1.59, -1.12], *P* < 0.001; β (95% CI): -0.01 [-0.99, -0.01], *P* < 0.001). BMI was negatively associated with lumbar QCT-BMD (β (95% CI): -1.60 [-2.25, -0.95], *P* < 0.001) and positively associated with total hip DXA-BMD (β (95% CI): 0.01 [0.01–0.01], *P* = 0.001). Males exhibited significantly higher lumbar QCT-BMD than females (β (95% CI): 17.37 [8.46–26.28], *P* < 0.001). Non-smokers had significantly greater lumbar QCT-BMD (β (95% CI): 13.19 [2.53–23.85], *P* = 0.016). Participants with hyperlipidemia had significantly lower total hip DXA-BMD (β (95% CI): -0.04 [-0.06, -0.01], *P* = 0.002), and non-diabetic individuals had a significantly reduced risk of vertebral fractures (OR (95% CI): 0.33 [0.17–0.63], *P* < 0.001) (**Table 3**).

**Table 3:** Multivariate regression analysis.

	Lumbar QCT-BMD		Lumbar DXA-BMD		Hip DXA-BMD		Vertebral fracture	
Variables	β (95%CI)	*P*	β (95%CI)	*P*	β (95%CI)	*P*	OR (95%CI)	*P*
Model 1								
TSHI	1.97 (-3.80 ~ 7.74)	0.504	-0.09 (-0.58 ~ 0.40)	0.707	-0.00 (-0.03 ~ 0.02)	0.837	0.98 (0.62 ~ 1.57)	0.943
TT4RI	-0.08 (-0.28 ~ 0.11)	0.410	0.00 (-0.01 ~ 0.02)	0.586	0.00 (-0.00 ~ 0.00)	0.083	1.01 (0.97 ~ 1.04)	0.686
TFQI	-4.26 (-18.22 ~ 9.69)	0.550	0.52 (-0.67 ~ 1.70)	0.394	0.04 (-0.02 ~ 0.10)	0.214	1.01 (0.33 ~ 3.14)	0.983
TT4 nmol l	0.07 (-0.12 ~ 0.25)	0.475	-0.01 (-0.02 ~ 0.01)	0.266	0.00 (-0.00 ~ 0.00)	0.588	1.00 (0.99 ~ 1.02)	0.842
TT3 nmol L	0.04 (-0.14 ~ 0.22)	0.676	-0.01 (-0.02 ~ 0.01)	0.272	-0.00 (-0.00 ~ 0.00)	0.632	1.02 (1.01 ~ 1.04)	0.008
TSH mIU L	0.80 (-0.17 ~ 1.77)	0.106	0.03 (-0.11 ~ 0.06)	0.538	-0.01 (-0.99 ~ -0.01)	0.032	0.91 (0.57 ~ 1.45)	0.699
FT4 pmol L	0.24 (-2.14 ~ 2.62)	0.843	-0.04 (-0.24 ~ 0.16)	0.706	-0.01 (-0.02 ~ 0.00)	0.244	1.06 (0.75 ~ 1.52)	0.731
FT3 pmol L	2.43 (-1.53 ~ 6.38)	0.231	-0.08 (-0.41 ~ 0.26)	0.659	0.01 (-0.01 ~ 0.03)	0.382	0.49 (0.15 ~ 1.57)	0.227
FT4/FT3	3.35 (-3.27 ~ 9.98)	0.322	-0.13 (-0.69 ~ 0.43)	0.650	-0.02 (-0.05 ~ 0.01)	0.233	0.49 (0.13 ~ 1.93)	0.308
Model2								
TSHI	0.05 (-4.85 ~ 4.95)	0.983	-0.09 (-0.59 ~ 0.40)	0.709	-0.00 (-0.03 ~ 0.02)	0.853	1.04 (0.65 ~ 1.66)	0.877
TT4RI	-0.06 (-0.23 ~ 0.10)	0.448	0.00 (-0.01 ~ 0.02)	0.580	0.00 (-0.00 ~ 0.00)	0.139	1.01 (0.97 ~ 1.04)	0.707
TFQI	-5.33 (-17.14 ~ 6.49)	0.377	0.51 (-0.68 ~ 1.70)	0.401	0.04 (-0.02 ~ 0.10)	0.231	1.09 (0.34 ~ 3.44)	0.886
TT4 nmol l	0.20 (0.04 ~ 0.35)	0.014	-0.01 (-0.03 ~ 0.01)	0.242	0.00 (-0.00 ~ 0.00)	0.362	1.00 (0.98 ~ 1.01)	0.845
TT3 nmol L	0.14 (-0.01 ~ 0.30)	0.074	-0.01 (-0.02 ~ 0.01)	0.259	0.00 (-0.00 ~ 0.00)	0.854	1.02 (1.01 ~ 1.03)	0.028
TSH mIU L	1.02 (0.20 ~ 1.84)	0.015	0.03 (-0.11 ~ 0.06)	0.532	-0.01 (-0.99 ~ -0.01)	0.036	0.90 (0.54 ~ 1.52)	0.698
FT4 pmol L	-1.54 (-3.58 ~ 0.49)	0.138	-0.03 (-0.23 ~ 0.18)	0.802	-0.01 (-0.02 ~ 0.00)	0.051	1.09 (0.77 ~ 1.54)	0.625
FT3 pmol L	3.63 (0.27 ~ 7.00)	0.035	-0.07 (-0.41 ~ 0.27)	0.670	0.00 (-0.01 ~ 0.02)	0.579	0.49 (0.16 ~ 1.53)	0.219
FT4/FT3	11.24 (5.50 ~ 16.99)	<0.001	-0.18 (-0.76 ~ 0.39)	0.531	-0.00 (-0.03 ~ 0.03)	0.907	0.44 (0.12 ~ 1.64)	0.222
Age	-1.32 (-1.55 ~ -1.09)	<0.001	0.01 (-0.01 ~ 0.03)	0.380	-0.01 (-0.99 ~ -0.01)	<0.001	1.02 (1.00 ~ 1.04)	0.075
BMI kg m2	-1.54 (-2.19 ~ -0.90)	<0.001	-0.01 (-0.07 ~ 0.06)	0.814	0.01 (0.01 ~ 0.01)	<0.001	1.06 (0.99 ~ 1.12)	0.088
Gender								
Female	Ref.		Ref.		Ref.		1.00 (Reference)	
Male	13.42 (6.25 ~ 20.58)	<0.001	-0.04 (-0.76 ~ 0.68)	0.913	0.06 (0.03 ~ 0.10)	<0.001	0.52 (0.24 ~ 1.12)	0.094
Model3								
TSHI	-0.76 (-5.72 ~ 4.20)	0.765	-0.13 (-0.64 ~ 0.37)	0.603	0.00 (-0.02 ~ 0.03)	0.902	1.23 (0.74 ~ 2.04)	0.431
TT4RI	-0.03 (-0.20 ~ 0.14)	0.739	0.01 (-0.01 ~ 0.02)	0.500	0.00 (-0.00 ~ 0.00)	0.218	1.00 (0.97 ~ 1.04)	0.807
TFQI	-5.09 (-16.89 ~ 6.71)	0.399	0.47 (-0.73 ~ 1.67)	0.444	0.03 (-0.03 ~ 0.09)	0.279	1.28 (0.38 ~ 4.24)	0.690
TT4 nmol l	0.18 (0.02 ~ 0.35)	0.027	-0.01 (-0.03 ~ 0.01)	0.245	0.00 (-0.00 ~ 0.00)	0.999	0.99 (0.98 ~ 1.01)	0.361
TT3 nmol L	0.12 (-0.03 ~ 0.28)	0.124	-0.01 (-0.03 ~ 0.01)	0.211	-0.00 (-0.00 ~ 0.00)	0.993	1.02 (1.01 ~ 1.04)	0.018
TSH mIU L	0.89 (0.07 ~ 1.71)	0.034	0.03 (-0.12 ~ 0.05)	0.451	-0.01 (-0.99 ~ -0.01)	0.053	0.91 (0.59 ~ 1.41)	0.682
FT4 pmol L	-1.73 (-3.78 ~ 0.33)	0.100	-0.02 (-0.23 ~ 0.19)	0.845	-0.01 (-0.02 ~ 0.00)	0.087	1.12 (0.77 ~ 1.62)	0.544
FT3 pmol L	3.69 (0.31 ~ 7.07)	0.033	-0.04 (-0.38 ~ 0.30)	0.822	0.01 (-0.01 ~ 0.02)	0.427	0.45 (0.13 ~ 1.53)	0.202
FT4/FT3	12.08 (6.18 ~ 17.98)	<0.001	-0.12 (-0.72 ~ 0.48)	0.693	-0.00 (-0.03 ~ 0.03)	0.914	0.33 (0.08 ~ 1.38)	0.128
Age	-1.36 (-1.59 ~ -1.12)	<0.001	0.01 (-0.01 ~ 0.03)	0.443	-0.01 (-0.99 ~ -0.01)	<0.001	1.02 (1.00 ~ 1.04)	0.106
BMI kg m2	-1.60 (-2.25 ~ -0.95)	<0.001	-0.00 (-0.07 ~ 0.06)	0.933	0.01 (0.01 ~ 0.01)	0.001	1.05 (0.98 ~ 1.12)	0.135
Gender								
Female	Ref.		Ref.		Ref.		1.00 (Reference)	
Male	17.37 (8.46 ~ 26.28)	<0.001	-0.01 (-0.91 ~ 0.90)	0.985	0.03 (-0.01 ~ 0.08)	0.173	0.38 (0.14 ~ 1.01)	0.052
Cigarettes								
Yes	Ref.		Ref.		Ref.		1.00 (Reference)	
No	13.19 (2.53 ~ 23.85)	0.016	0.13 (-0.96 ~ 1.21)	0.819	-0.03 (-0.09 ~ 0.02)	0.260	0.94 (0.31 ~ 2.87)	0.920
Alcohol								
Yes	Ref.		Ref.		Ref.		1.00 (Reference)	
No	-9.11 (-23.31 ~ 5.08)	0.209	-0.25 (-1.69 ~ 1.20)	0.737	-0.04 (-0.11 ~ 0.03)	0.311	0.90 (0.17 ~ 4.71)	0.902
Hypertension								
Yes	Ref.		Ref.		Ref.		1.00 (Reference)	
No	-4.28 (-9.19 ~ 0.64)	0.089	0.17 (-0.33 ~ 0.67)	0.509	-0.01 (-0.03 ~ 0.02)	0.523	1.21 (0.74 ~ 1.96)	0.451
Hyperlipidemia								
No	Ref.		Ref.		Ref.		1.00 (Reference)	
Yes	-1.84 (-6.66 ~ 2.99)	0.456	-0.45 (-0.94 ~ 0.04)	0.074	-0.04 (-0.06 ~ -0.01)	0.002	1.07 (0.66 ~ 1.71)	0.794
Diabetes								
Yes	Ref.		Ref.		Ref.		1.00 (Reference)	
No	2.63 (-3.79 ~ 9.05)	0.423	0.18 (-0.47 ~ 0.83)	0.590	-0.02 (-0.05 ~ 0.01)	0.234	0.33 (0.17 ~ 0.63)	<0.001
Cardiovascular disease								
Yes	Ref.		Ref.		Ref.		1.00 (Reference)	
No	4.32 (-2.75 ~ 11.39)	0.232	0.11 (-0.61 ~ 0.83)	0.766	-0.00 (-0.04 ~ 0.03)	0.927	0.57 (0.28 ~ 1.19)	0.134

### 3.6 Subgroup analysis and interaction effects

**Figure 1:**
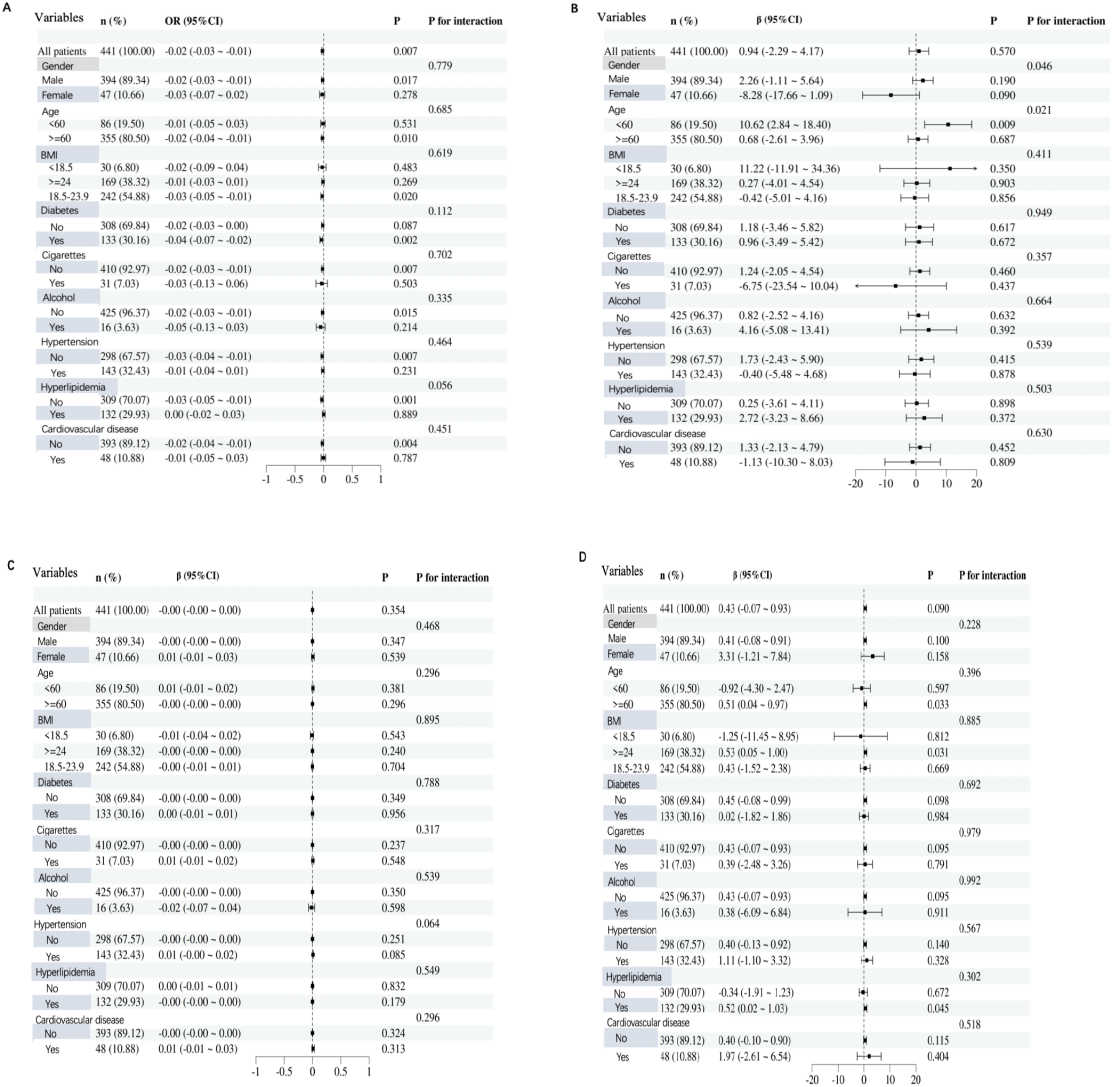
Subgroup analysis: **(A)** In the association between TT3 and vertebral fracture risk, no significant interaction effects were observed for gender, age, BMI, smoking, alcohol consumption, diabetes, hypertension, hyperlipidemia, or history of cardiovascular disease across subgroups; **(B)** For the association between FT4/FT3 and lumbar QCT-BMD, significant interactions were found by gender (*P* for interaction = 0.046) and age (*P* for interaction = 0.021). Among individuals aged 50~60 years, the FT4/FT3 ratio was significantly associated with lumbar QCT-BMD (*P* = 0.009; β (95% CI): 10.62 [2.84–18.40]); **(C)** In the association between FT3 and lumbar QCT-BMD, no significant interaction effects were detected across any of the predefined subgroups; **(D)** No significant interaction effects were observed in the association between TSH and lumbar QCT-BMD across subgroups.

**Figure 2:**
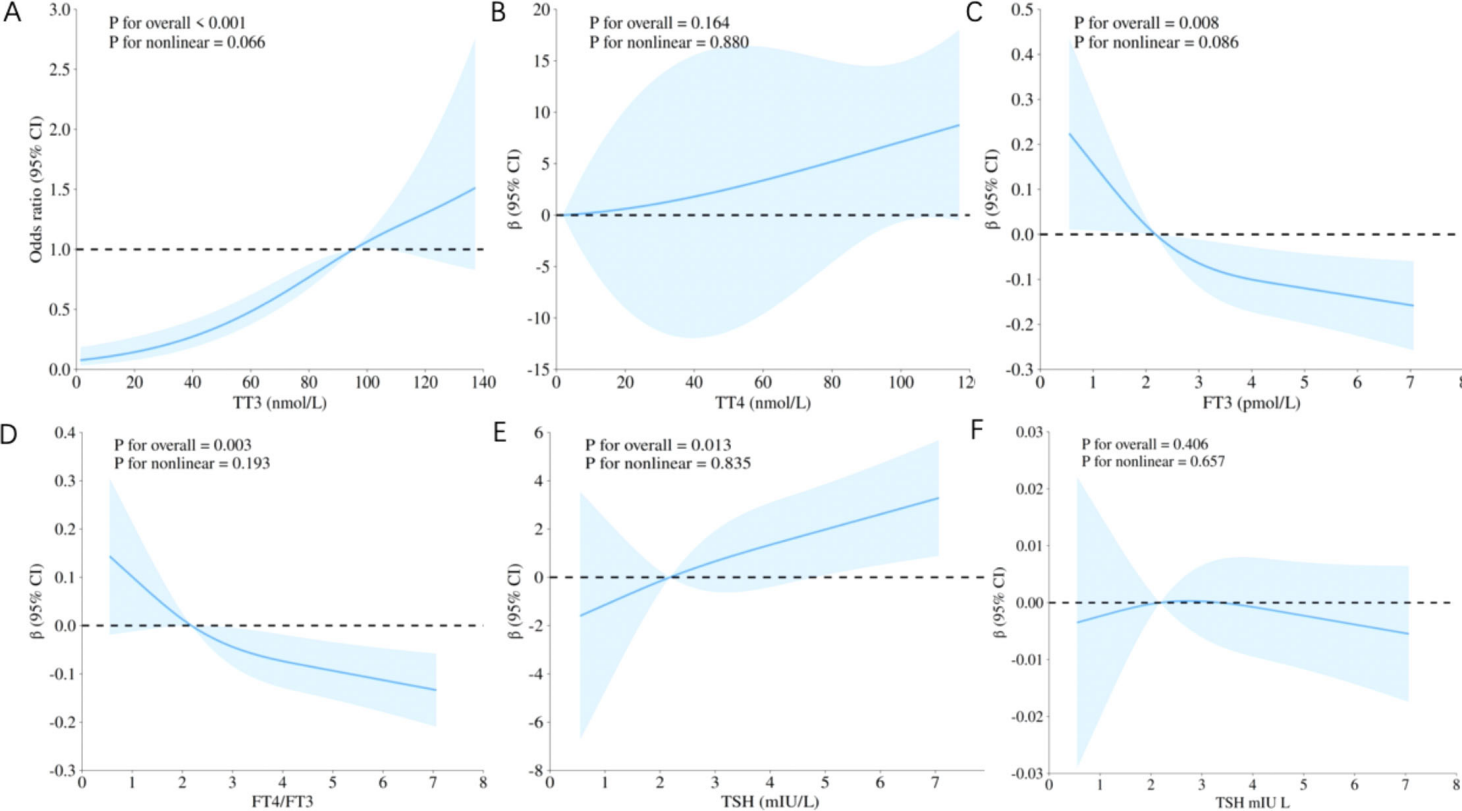
RCS analysis: **(A)** TT3 levels were positively associated with an increased risk of vertebral fractures (*P* < 0.001), but did not exhibit significant non-linearity (*P* = 0.066); **(B)** No significant association was observed between TT4 and lumbar QCT-BMD; **(C)** FT3 was significantly inversely associated with lumbar QCT-BMD (*P* = 0.008), with no evidence of a non-linear relationship (*P* = 0.086); **(D)** FT4/FT3 ratio showed a significant inverse association with lumbar QCT-BMD (*P* = 0.003), and this association remained linear (*P* = 0.193); **(E)** TSH was significantly positively associated with lumbar QCT-BMD (*P* = 0.013), and the dose-response relationship was linear (*P* = 0.835); **(F)** No significant association was found between TSH and total hip DXA-BMD.

Subgroup analyses were performed with stratification by gender, age, BMI, diabetes, hypertension, hyperlipidemia, cardiovascular disease, smoking, and alcohol consumption. In the association between FT4/FT3 ratio and lumbar QCT-BMD, significant interaction effects were observed for both gender (*P* for interaction = 0.046) and age (*P* for interaction = 0.021) (**Figure 1B**). Among individuals younger than 60 years old, a higher FT4/FT3 ratio was significantly associated with increased lumbar QCT-BMD (*P* = 0.009; β (95% CI): 10.62 [2.84–18.40]) (**Figure 2B**). No statistically significant interactions were detected for the other covariates across subgroups (**Figures 1A, C, D**).

### 3.7 Restricted cubic spline analysis

Higher TT3 levels were positively associated with an increased risk of vertebral fractures (*P* < 0.001), and the relationship did not exhibit significant non-linearity (*P* = 0.066) (**Figure 2A**). No significant association was observed between TT4 and lumbar QCT-BMD (**Figure 2B**). In RCS analysis, FT3 was significantly inversely associated with lumbar QCT-BMD (*P* = 0.008), with no evidence of a non-linear relationship (*P* = 0.086) (**Figure 2C**). Similarly, the FT4/FT3 ratio showed a significant inverse association with lumbar QCT-BMD (*P* = 0.003), and no evidence of a non-linear relationship (*P* = 0.193) (**Figure 2D**). TSH was significantly positively associated with lumbar QCT-BMD (*P* = 0.013), and no obvious non-linear relationship (*P* = 0.835) (**Figure 2E**). In contrast, no significant association was found between TSH and total hip DXA-BMD (**Figure 2F**).

## Data Availability

The raw data supporting the conclusions of this article will be made available by the authors, without undue reservation.
